# Prevalence of gastrointestinal parasites in cattle in Indonesia: A meta-analysis and systematic review

**DOI:** 10.14202/vetworld.2024.2675-2687

**Published:** 2024-11-28

**Authors:** Vika Ichsania Ninditya, Fitrine Ekawasti, Joko Prastowo, Irkham Widiyono, Wisnu Nurcahyo

**Affiliations:** 1 Department of Parasitology, Graduate Student of Veterinary Sciences, Faculty of Veterinary Medicine, Universitas Gadjah Mada, Yogyakarta, Indonesia; 2 Department of Parasitology, Faculty of Veterinary Medicine, Universitas Gadjah Mada, Yogyakarta, Indonesia; 3 Research Center for Veterinary Science, Research Organization for Health, National Research and Innovation Agency, Bogor, West Java, Indonesia; 4 Department of Internal Medicine, Faculty of Veterinary Medicine, Universitas Gadjah Mada, Yogyakarta, Indonesia

**Keywords:** cattle, gastrointestinal, Indonesia, parasites, prevalence

## Abstract

**Background and Aims:**

Gastrointestinal parasites (GIPs) pose a major health challenge for cattle in Indonesia. GIP infections affect the production and reproductive performance of cattle, resulting in economic losses. However, the prevalence and distribution of infections have not been comprehensively profiled at the national level. This study aimed to estimate the prevalence of GIP infections in cattle in Indonesia.

**Materials and Methods:**

Overall, 667 articles were identified from six databases in English and Bahasa Indonesia. After removing duplicates and screening titles and abstracts based on the inclusion criteria (i.e., GIP prevalence in cattle in Indonesia), 67 articles were included in the data review. Data were pooled using a random-effects model in STATA software. Heterogeneity was tested using Cochran’s Q-value and I^2^ statistics, whereas publication bias was assessed using Egger’s regression test.

**Results:**

The overall pooled prevalence of GIP in Indonesia was 46% (95% confidence interval 37%–55%), with a total population of 17,278 cattle screened. The I^2^ value was 99.59%, Cochran’s Q-value was 15,957.25, and p = 0.001. The results of the regional meta-analysis based on the provinces in the three zones of Indonesia showed estimated prevalence rates of 54.0%, 52.7%, and 53.7% in Western, Central, and Eastern Indonesia, respectively. The parasite with the highest prevalence was *Eimeria* spp. (37.7%), followed by nematodes (34.4%) and trematodes (*Fasciola* spp., 21.4%).

**Conclusion:**

The findings reveal a high prevalence of GIPs in cattle across Indonesia, with significant variability across regions and parasite types. *Eimeri*a spp., nematodes, and trematodes represent the most prevalent infections and underscore the urgent need for region-specific control strategies, including improved livestock management practices, routine screening, and integrated parasitic control programs.

## Introduction

Gastrointestinal parasites (GIPs) continue to pose challenges to livestock farming, particularly cattle farming. Although many of these parasites do not manifest with overt clinical symptoms, their presence can significantly impact economic growth by hindering optimal growth in cattle [1, 2]. The presence of GIPs in cattle causes decreased weight gain, anemia, delayed growth, tissue damage, and reduced overall productivity [3]. Many cattle farms in Indonesia remain traditional and are located near the owners’ houses [4]. The indiscriminate use of dewormers without proper veterinary supervision results in resistance among parasitic organisms, which poses a significant challenge to effective parasite control [5, 6]. The bovine gastrointestinal tract is susceptible to parasitic infections. Among these, nematodes, such as *Trichostrongylus, Oesophagostomum, Toxocara vitulorum*, and *Strongyloides* spp.; cestodes, such as *Moniezia* spp.; trematodes, such as *Fasciola* spp. and *Paramphistomum* spp.; and protozoan infections, particularly those caused by *Eimeria* spp., are prevalent in cattle [5]. Parasitic infections adversely affect livestock health and productivity. These effects can include reduced reproductive performance, decreased appetite, weight loss, decreased milk production, and increased susceptibility to other diseases [1, 3]. Epidemiological surveys of GIPs are important to avoid economic losses by informing the implementation of effective control efforts [7].

The prevalence of GIPs in cattle varies across regions in Indonesia, with differing rates of infection and parasite diversity [5, 6, 8]. These parasites cause various clinical symptoms and health issues in cattle, affecting their overall well-being and productivity, for example, gastrointestinal nematode parasites such as *Trichuris* and *Capillaria* in Indonesia [6]. Furthermore, trematode infections, including those by *Fasciola gigantica* and *Paramphistomum* spp., have been documented in cattle in various regions of Indonesia [9, 10]. These infections can cause liver damage, reduced productivity, and other health issues in cattle, thereby affecting the economic viability of livestock farming. In addition, the identification of *Eurytrema* spp. trematodes in the pancreatic tracts of domestic ruminants in Indonesia highlight the diversity of parasitic infections that can affect cattle health [11]. Effective management of GIP is crucial for enhancing livestock production, particularly in the beef industry [4, 12]. Therefore, regular epidemiological monitoring is necessary to identify and assess parasite infection levels [13]. This information is essential for developing deworming strategies that ensure optimal animal health and productivity [14]. Although numerous studies Nurcahyo *et al*. [6], Ekawasti *et al*. [15] have reported on parasites affecting cattle in Indonesia, most were limited to specific regions, and some were only accessible in local journals, hindering their global reach and accessibility to researchers worldwide.

This meta-analysis aimed to provide a comprehensive understanding of the prevalence of GIPs on a national scale.

## Materials and Methods

### Ethical approval

The systematic review adhered to the Preferred Reporting Items for Systematic Reviews and Meta-Analyses (PRISMA) criteria [16].

### Study period and location

The search period started in December 2023 until April 2024 and the analysis of the data started in May till June 2024. The data was processed at Department of Parasitology, Faculty of Veterinary Medicine, Universitas Gadjah Mada.

### Search strategy and study protocol

We comprehensively searched six databases (PubMed, ScienceDirect, Scopus, Google Scholar, Sinta, and Garuda [Indonesian Literature Database]) for articles indexed from December 21, 2023, to May 10, 2024. A search was conducted on the Garuda (https://garuda.kemdikbud.go.id/) and Sinta (https://sinta.kemdikbud.go.id/) websites in Indonesia. Keywords were strategically combined using “AND” and “OR” operators to enhance search precision. The keywords used are listed in Table-1.

**Table-1:** Detailed search strategies used in this review.

Database	Scope	Keywords
PubMed	All files	(Cattle) OR (cattle[MeSH Terms]) OR (bovine[MeSH Terms]) OR (cow[MeSH Terms]) OR (ruminant[MeSH Terms]) AND (gastrointestinal parasites) OR (endoparasite) OR (helminth[MeSH Terms]) OR (protozoa[MeSH Terms]) OR (intestinal parasites[MeSH Terms]) OR (cestodes[MeSH Terms]) OR (nematodes[MeSH Terms]) OR (trematode[MeSH Terms]) OR (strongyle[MeSH Terms]) OR (eimeria[MeSH Terms]) OR (coccidiosis[MeSH Terms]) AND (indonesia)
Google Scholar	All in title	allintitle: Indonesia cattle cow OR bovine OR endoparasites OR cestodes OR ruminants OR parasites OR helminth OR nematodes OR protozoan OR eimeria OR fasciola OR gastrointestinal OR strongyle OR livestock
ScienceDirect	Keywords	Indonesia cattle cow OR bovine OR endoparasites OR cestodes OR ruminants OR parasites OR helminth OR nematodes OR protozoan OR eimeria OR fasciola OR gastrointestinal OR strongyle OR livestock
Scopus	Keywords, title, abstract	“Gastrointestinal parasites” OR “Endoparasite” OR “Helminth” OR “protozoa” OR “intestinal parasites” OR “cestodes” OR “nematodes” OR “Trematodes” OR “Strongyle” OR “strongyloides” OR “Eimeria” OR “Fasciola” AND “Cattle” OR “Bovine” OR “Cow” OR “ruminants” AND “Indonesia”
Garuda	Title and keywords	Prevalensi (Indonesian), Parasit (Indonesian), Sapi (Indonesian); AND Prevalensi (Indonesian), trematoda (Indonesian), sapi (Indonesian); AND Prevalensi (Indonesian), nematoda (Indonesian), Sapi (Indonesian).
Sinta	All in title	Helminth Cattle AND parasit sapi (Indonesian)

### Inclusion and exclusion criteria

The collected articles were selected by authors VIN and FE based on a review of the titles and were then further screened based on the abstracts and titles. The unapproved articles were discussed with authors JP and WN. Only articles published in journals or proceedings were included in this analysis. The inclusion criterion was research on the prevalence of GIPs in Indonesian cattle. The articles included information about the number of samples and positive results. The exclusion criteria were research records marked before 2000, irrelevant study subject, no prevalence information, no report of gastrointestinal parasites, not in cattle, and unclear results.

### Quality assessment

Data were extracted using a standardized protocol to obtain the study location, cattle breed, diagnostic methodology, sample size, number of positive cases, and prevalence. Each article was meticulously evaluated for quality using the Grading of Recommendations, Assessment, Development, and Evaluation criteria, which considered factors such as random sampling, precise sample location with complete latitude and longitude coordinates, sample size >200 individuals, clearly defined detection methods, and risk factor analysis. The score for each article ranged from 1 to 5. Quality assessment encompassed: 1. Random sampling. 2. Clearly defined sampling locations, including latitudes and longitudes. 3. The total number of samples exceeded 200. 4. A clear detection method was employed. 5. Risk factor analysis was conducted. If only one criterion was met, a score of one was assigned; if all criteria were met, a score of five was assigned.

### Statistical analysis

Data were imported into Microsoft Excel 2021 version 2410 (Microsoft Office, Washington, USA) and analyzed using STATA BE 18 (StataCorp, https://www.stata.com/) for meta-analysis and statistical analyses. We used the methods for prevalence estimation, heterogeneity estimation, and significance estimation as described by Abbas *et al*. [17] and DerSimonian and Laird [18]. The effect size for prevalence was processed using raw transformation, whereas the pooled prevalence was calculated using a 95% confidence interval (CI). Subsequently, a meta-analysis was performed using the random-effects DerSimonian–Laird method. The Cochran (Q) statistic was used to determine the presence of heterogeneity in prevalence between studies. In addition, the I^2^ statistic was calculated, with values >50% indicating high heterogeneity. Publication bias was evaluated using funnel plots to visualize the relationship between the standard error and the logarithm of the effect’s size. Egger’s linear regression test was conducted with p < 0.05 considered statistically significant, suggesting asymmetry in the results. Random-effect models were used for analyses according to province and specific parasites.

## Results

### Article selection

**Figure-1:**
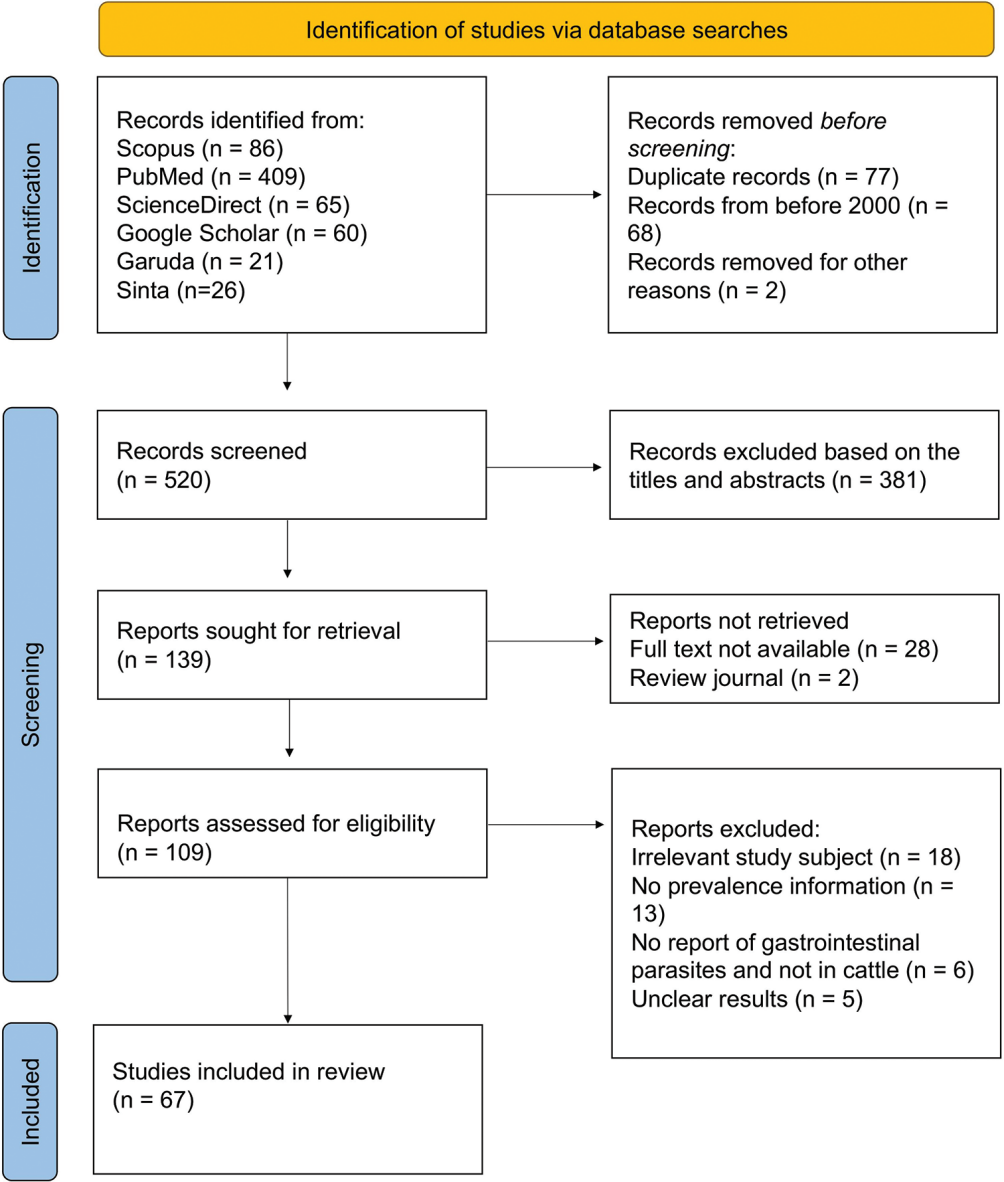
Preferred reporting items for systematic reviews and meta-analyses: Flow diagram illustrating selection methodology.

In total, 667 scientific publications were identified using EndNote. After removing 77 duplicates and 68 articles published before 2000, 520 articles were screened based on their titles and abstracts. An additional 381 articles were excluded because they did not meet the inclusion criteria, such as those containing parasites other than those in the gastrointestinal tract. Figure-1 shows the detailed PRISMA flow chart and selection methodology.

Of the remaining 139 articles, 28 did not have accessible full texts, and two were review journals, leaving 109 articles for further review. Some of the remaining articles were unsuitable for study inclusion due to a lack of information on the prevalence of non-GIPs in cattle and unclear methods or results. Finally, 67 articles were included in the meta-analysis (Table-2) [1-12, 15, 19–73].

**Table-2:** Studies included in this meta-analysis on the prevalence of bovine gastrointestinal parasites in Indonesia.

No.	Breed	Method	No. tested	Overall positive	Percentage	Location	QA	References
1	Madura cattle	Sedimentation and modified Fülleborn’s floatation method	500	357	71.40	10 districts at Bangkalan, Madura, East Java	4	[1]
2	Cattle	Sugar flotation and sedimentation	394	153	38.83	West Java	2	[2]
3	Aceh cattle	Modification of sedimentation	103	28	27.18	Banda Aceh City, Aceh, Saudi Arabia	1	[3]
4	Various cattle	The double centrifugation method	102	44	43.10	Bondowoso Regency, East Java, Indonesia	1	[4]
5	Madura cattle	Whitlock sedimentation methods.	400	55	13.75	Pakong, Pasean, Batumarmar, and Waru Districts, Madura Regency, East Java	4	[5]
6	Various cattle	Whitlock and flotation methods	335	81	24.20	North Sumatera, West Sumatera, Yogyakarta, East Java, Central Sulawesi, South-East Sulawesi, West Sulawesi, North Kalimantan, Central Kalimantan, West Nusa Tenggara, East Nusa Tenggara, North Maluku, Papua		[6]
7	Dairy cattle	Flukefinder® kit and simple sedimentation technique	400	66	16.50	Boyolali Regency, Central Java.	3	[7]
8	Various cattle	Sedimentation and floatation methods	100	35	35.00	Jombang Regency, East Java	2	[8]
9	Various cattle	Sedimentation and Parfitt–Banks methods	100	50	50	Progo River, Yogyakarta	2	[9]
10	Buffalo and cattle	Boray’s modified sedimentation method	199	96	48.24	East Lampung and Lebak Provinces	3	[10]
11	Beef cattle	Sedimentation and flotation methods	100	24	24.00	Lamongan District, East Java	1	[12]
12	Cattle	Sugar floatation and PCR	817	534	65.40	North Sumatera, West Sumatera, Bangka Belitung, West Java, Central Java, Yogyakarta, East Java, Central Sulawesi, Southeast Sulawesi, West Sulawesi, South Sulawesi, Central Kalimantan, East Kalimantan, the West Nusa Tenggara Islands, the East Nusa Tenggara Islands, North Maluku, Papua, and West Papua.	3	[15]
13	Bali cattle	Sedimentation and floating methods	100	62	62	Lopok Subdistrict, Sumbawa District, West Nusa Tenggara	2	[19]
14	Crossbreed and Bali cattle	Modified Whitlock and sedimentation methods	100	74	74	East Kalimantan and Riau	3	[20]
15	Various cattle	Flotation and PCR	171	43	25.10	Banten, Lampung, and West Java	2	[21]
16	Beef cattle	Modified Danish Bilharziasis Laboratory technique for sedimentation	480	252	52.50	Prafi district, Manokwari Regency, West Papua, Indonesia	5	[22]
17	Madura cattle	Modified sugar flotation and polymerase chain reaction	100	21	21	Kamal Subdistrict, Madura, East Java	3	[23]
18	Madura cattle	Sugar centrifugal flotation and polymerase chain reaction	183	60	32.80	Socah and Kamal subdistricts, Madura, East Java		[24]
19	Cattle	PCR	120	36	30	Sulawesi South, West, Central, and Gorontalo	1	[25]
20	Beef cattle	PCR	108	108	100.00	Kamal and Socah subdistricts, Bangkalan district, Madura, East Java	1	[26]
21	Various cattle	Visual observation	585	147	25.12	Boyolali District, Central Java	3	[27]
22	Cattle	Simple sedimentation and Fülleborn floatation methods	75	57	76.00	Magelang District, Central Java	2	[28]
23	Bali cattle	Flotation method	62	15	24.19	Denpasar City, Bali	1	[29]
24	Cattle	Whitlock flotation method	91	42	46.15	Napis village, Bojonegoro Regency, East Java.	1	[30]
25	Bali cattle	Flotation method	100	25	25.00	Nusa Penida Regency, Bali	1	[31]
26	Bali cattle	Flotation method	55	29	52.73	Tabanan Regency, Bali	1	[32]
27	Bali cattle	Flotation method	182	129	70.88	Badung Regency, Bali	1	[33]
28	Various cattle	Sedimentation test	314	153	48.70	Jember district, East Java	3	[34]
29	Dairy cattle	Flotation and sedimentation processes	36	24	66.67	Blitar Regency, East Java	1	[35]
30	Various cattle	Whitlock sedimentation methods	130	48	36.92	Cibungbulang District, Bogor, West Java	1	[36]
31	Bali cattle	Flotation method	288	91	31.60	Gianyar and Badung Regency, Denpasar, Bali.	3	[37]
32	Bali cattle	Sedimentation and floating methods	100	65	65	Denpasar City, Bali	1	[38]
33	Cattle	Whitlock method, sugar floatation, and polymerase chain reaction	289	151	52.30	11 districts in Central Java, 2 in East Java, and 1 in Yogyakarta	4	[39]
34	Cattle	Flotation and sedimentation processes	151	93	61.59	East Java, Banten, West Nusa Tenggara, Bali	3	[40]
35	Cattle	Sugar floatation and PCR	70	45	64.28	Biak and Serui Regency, Papua, Indonesia	1	[41]
36	Bali cattle	Flotation method	100	21	21.00	Badung Regency, Bali	1	[42]
37	Various cattle	Modified McMaster technique	2150	1550	72.07	Riau, South Sumatera, Banten, the Yogyakarta Special Region, East Java, Bali, West Nusa Tenggara, Central Kalimantan, and South Sulawesi	5	[43]
38	Aceh cattle	Modified Knott’s technique and adult parasites	150	70	46.60	Banda Aceh slaughterhouse, Aceh, Saudi Arabia	1	[44]
39	Cattle	Floating method	120	70	58.33	Keleyan Village, Socah subdistrict, Bangkalan District, East Java	3	[45]
40	Aceh cattle	Flotation method	50	22	44.00	Aceh Besar Regency, Aceh	1	[46]
41	Bali cattle	Sedimentation and flotation methods	50	36	72.00	Bolo Subdistrict, Bima District, West Nusa Tenggara	1	[47]
42	Cattle	Flotation method	50	15	30.00	Kendari City, Southeast Sulawesi	1	[48]
43	Madura cattle	Flotation method	100	58	58.00	Geger, Bangkalan, Madura, East Java	1	[49]
44	Bali cattle	Sedimentation method	290	16	5.51	Mengwi District, Badung Regency, Bali	2	[50]
45	Dairy Cattle	Flotation method	75	15	20.00	Jember Regency, East Java	2	[51]
46	Cattle	Flotation and sedimentation processes	63	55	87.30	Progo River, Yogyakarta	1	[52]
47	Cattle	Whitlock and sedimentation methods	134	18	13.43	Lamongan, Gresik Regency, East Java	1	[53]
48	Cattle	Sedimentation and Fülleborn’s flotation method	41	30	73.17	Surabaya, East Java	1	[54]
49	Cattle	Sedimentation and liver examination	100	27	27.00	West Java	1	[55]
50	Bali Cattle	Floatation and sedimentation processes	369	212	57.45	Prafi district, Manokwari regency, West Papua, Indonesia	3	[56]
51	Beef cattle	Qualitative flotation method	120	46	38.33	Udapi Hilir Subdistrict, Manokwari Regency, West Papua	1	[57]
52	Various cattle	Qualitative flotation and the Baermann technique	633	221	34.91	Central Java	4	[58]
53	Cattle	Flotation method	144	25	17.36	Palang subdistrict, Tuban Regency, East Java	1	[59]
54	Bali cattle	Sedimentation method	100	27	27.00	Desa Sobangan, Mengwi, Badung, Bali	1	[60]
55	Aceh cattle	Flotation method	105	85	80.90	Mesjid Raya District, Aceh Besar, Aceh, Saudi Arabia	3	[61]
56	Beef cattle	Kato–Katz method	70	61	87.14	Slaughterhouse in Palu City, Central Sulawesi	0	[62]
57	Cattle	Sedimentation and flotation methods	100	40	40.00	Magetan Regency, East Java	2	[63]
58	Cattle	Sugar flotation	109	87	79.82	Tangerang district, Banten	3	[64]
59	Dairy cattle	Quantitative method	400	179	44.75	KPBS Pangalengan, South Bandung District, West Java	4	[65]
60	Bali cattle	Sedimentation and flotation methods	290	27	9.31	Sobangan Breeding Center, Badung, Bali	3	[66]
61	Beef cattle	Sedimentation and flotation methods	100	100	100.00	Siak Sri Indrapura, Riau	3	[67]
62	Cattle	Sedimentation method	40	35	87.50	Klumpang Kebon Village, Hamparan Perak District, Malaysia	1	[68]
63	Cattle	Floatation and sedimentation methods	2720	639	23.49	Jambi	2	[69]
64	Various cattle	Liver necropsy	100	39	39.00	Batu and Pujon Districts, East Java	1	[70]
65	Beef cattle	McMaster and sedimentation	240	48	20	Bangkalan Regency, Madura, East Java.	2	[71]
66	Cattle	Liver necropsy	106	39	36.79	Lima Puluh Kota Regency, Indonesia	2	[72]
67	Cattle	Flotation method	157	49	31.21	Gayo Lues, East Aceh, Saudi Arabia	1	[73]

PCR=Polymerase chain reaction, QA=Quality assessment

### Meta-analysis

The collected articles were subsequently assigned a score according to the established criteria. Specifically, 32 articles received a score of 1, 12 received a score of 2, 14 received a score of 3, four received a score of 4, and two received a score of 5. The average article did not include related risk factors or geographical coordinates of the study (Table-1).

**Figure-2:**
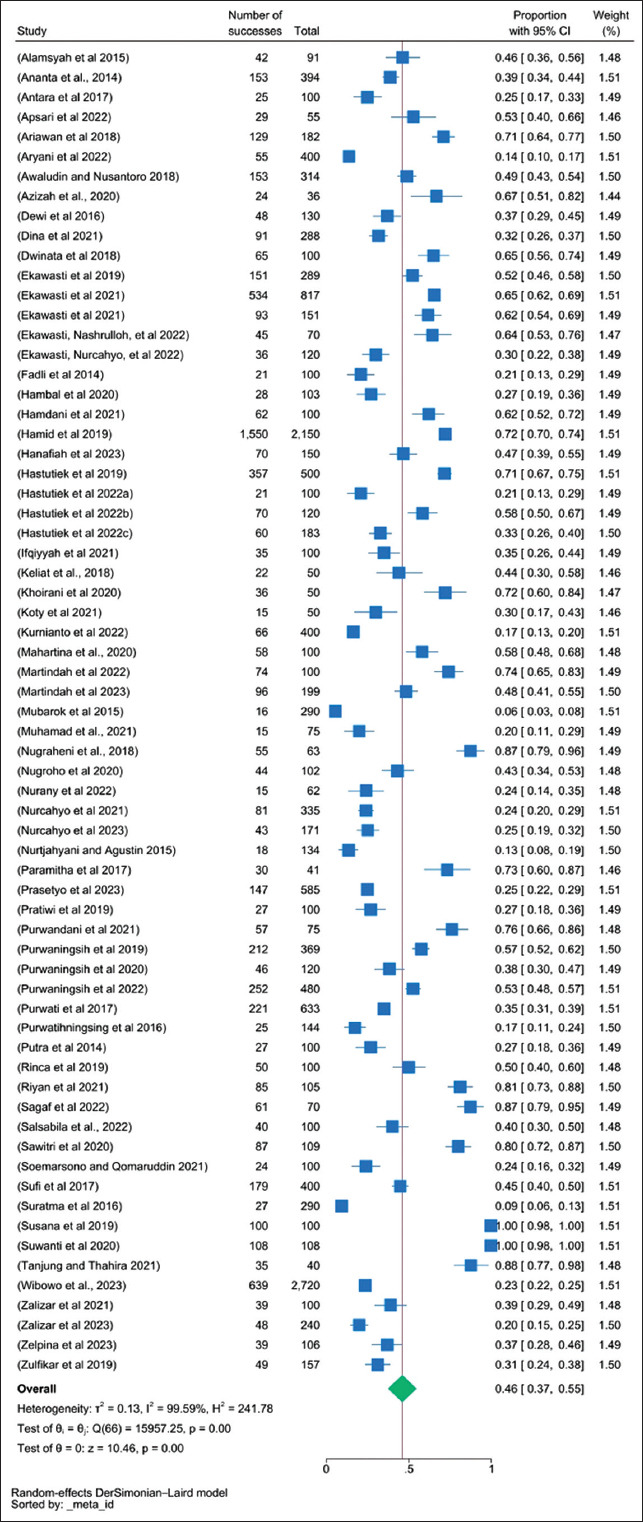
Forest plot of bovine gastrointestinal parasite infections in Indonesia using random-effect estimates.

**Figure-3:**
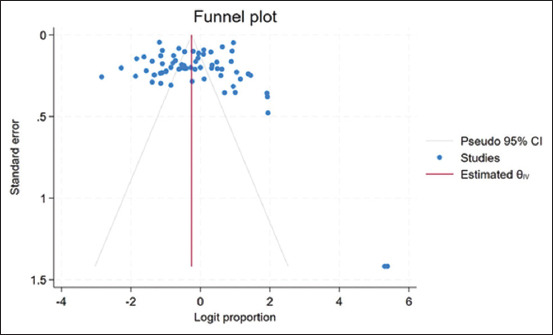
Funnel plot with 95% confidence interval of the logit proportion of gastrointestinal parasites in Indonesia.

The meta-analysis included 17,278 cattle, among which 7515 samples were positive. The prevalence rate was 46% (95% CI, 37%–55%). All analyses were performed for each species. The most common bovine GIP studied in Indonesia was *Fasciola*, followed by *Eimeria* and nematodes, such as *Trichuris* and *Strongyloides*. Based on the pooled prevalence analysis of each GIP species, the prevalence rate ranged between 0.5% and 37.1%, with the highest prevalence found for *Eimeria* spp., followed by nematodes such as *Trichuris* and *Strongyloides* (Table-3). Cochran’s Q test statistic was 15,957.25 at 66° of freedom (p < 0.001). This statistic was used to test statistical heterogeneity among pooled studies in the meta-analysis. In this case, the estimated between-study variance was 0.13, indicating that the variability in effect sizes was attributable to heterogeneity (99.59% based on the I^2^ statistic. The forest plot used to derive these estimates displays the individual study effects and pooled effects across studies, with Cochran’s Q calculated as the weighted sum of the squared differences between these effects (Figure-2). The funnel plot for studies published on bovine GIP in Indonesia (Figure-3) and its bias coefficient (b = 4.14, z = 4.94, p = 0.000) suggested no publication bias.

**Table-3:** Prevalence of various gastrointestinal parasites in cattle in Indonesia.

Gastrointestinal parasite	Number of studies	Total samples	Positive	Pooled estimate (%) based on 95% confidence interval	Heterogeneity			

					I^2^	τ^2^	Q-p	H^2^
Trematodes								
Amphistomes	1	199	67	34	-	-	-	
*Dicrocoelium*	1	40	1	3	-	-	-	
*Eurytrema*	1	50	2	4	-	-	-	
*Fasciola*	27	8225	1260	21.6 (16.8–26.3)	98.67	0.0146	1961.77	75.45
*Paramphistomum*	16	5587	644	16.2 (12–20.4)	98.1	0.0065	790.72	52.71
Cestodes								
*Moniezia* spp.	10	2060	85	4 (2.2–5.9)	81.24	0.0005	47.97	5.33
Nematode	5	856	227	34.4 (10.1–58.7)	98.62	0.0755	289.5	72.38
Strongyle-type	17	3475	1056	30.8 (22.8–38.7)	97.08	0.0268	547.1	34.19
*Trichuris* spp.	20	6116	141	3.3 (2.1–4.6)	91.53	0.0006	224.32	11.81
*Strongyloides*	19	5971	267	6.3 (4.6–8.0)	92.43	0.0009	237.74	13.21
*Toxocara vitulorum*	15	2372	159	6.3 (4–8.6)	91.56	0.0015	165.83	11.85
*Setaria* spp.	1	150	70	47	-	-	-	-
*Capillaria*	6	1629	47	2.4 (0.8–4)	83.75	0.0003	30.78	6.16
*Bunostomum* spp.	9	3815	54	2 (0.8–3.1)	72.18	0.0001	28.76	3.59
*Haemonchus* spp.	6	3609	118	9.9 (3.3–16.4)	95.98	0.0056	124.37	24.87
*Cooperia* spp.	5	4211	393	14.6 (4.1–25.1)	99.05	0.014	420.11	105.03
*Nematodirus* spp.	3	824	92	5.7 (2.3–13.7)	96.69	0.0048	60.33	30.17
*Mecistocirrus* spp.	3	177	12	5.6 (1.5–9.7)	29.42	0.0004	2.83	1.42
*Trichostrongylus* spp.	9	4612	231	8.6 (4.7–12.5)	97.03	0.003	268.94	33.62
*Chabertia* spp.	3	3453	89	5.1 (2.5–12.7)	97.58	0.0043	82.72	41.36
*Oesophogostomum* spp.	12	4619	172	5.9 (3.8–8.1)	94.9	0.001	215.88	19.63
*Ostertagia* spp.	5	4211	181	4.4 (2.2–6.6)	90.77	0.0005	43.32	1083
Ascarids	5	3707	50	3.3 (0.7–5.9)	94.53	0.0006	73.13	18.28
Protozoa								
*Eimeria*	24	9390	3451	37.3 (23.2–51.4)	99.69	0.1231	7423.53	322.76
*Blastocystis*	4	800	182	31 (-27.8–89.8)	99.94	0.3597	4778.96	1592.99
*Entamoeba*	4	755	109	27.9 (0.5–60.7)	99.65	0.1115	860.32	286.77
*Balantidium*	4	755	60	6.1 (1.6–10.6)	83.34	0.0016	18	6
*Giardia*	2	209	16	7.6 (4–11.2)	0	0	0.12	1
*Cryptosporidium*	4	1103	7	0.5 (0.1–0.9)	0	0	1.48	1
*Buxtonella*	1	40	23	-	-	-	-	-

### Analysis of bovine GIP

Five trematode species were identified: Amphistomes, *Fasciola, Eurytrema, Paramphistomum*, and *Dicrocoelum*. Only one cestode species, *Moniezia* spp., has been previously reported. The gastrointestinal nematode species exhibited the greatest diversity, with 15 distinct species identified (Table-3). Most nematode species were of the strongyle type. However, some studies did not perform species-level identification.

### Distribution of bovine GIPs according to location in Indonesia

**Figure-4:**
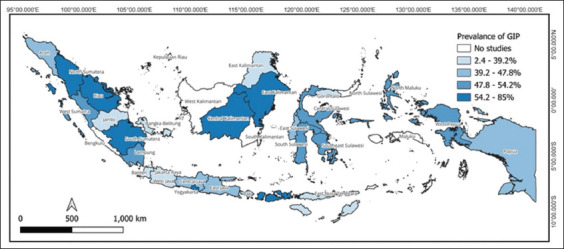
Distribution of gastrointestinal parasite prevalence across Indonesia [Source: The map was generated using QGIS 3.32.2, and the data of the map were extracted from https://diva-gis.org/data.html].

Regarding geographical distribution, most GIP-related studies in Indonesia have been conducted in the Western region, followed by the Central region. Figure-4 illustrates the distribution of GIP prevalence in Indonesia. Only three provinces in eastern Indonesia have reported GIP prevalence in cattle. East Java, in particular, has contributed the highest number of studies, 25 with a total reported population of 3693 cattle, indicating a pooled prevalence of 44.1% (95% CI, 27.8%–60.4%). Other regions exhibited varying prevalence rates, ranging from 28% to 85.7% (Table-4).

**Table-4:** Pooled prevalence of gastrointestinal parasites according to province in the Western, central, and Eastern regions of Indonesia.

Province	No. of studies	Total samples	Positive samples	Pool prevalence based on 95% confidence interval	Heterogenicity
Western Indonesia					
Aceh	5	565	254	46.1 (25.4–66.7)	96.59
Jambi	1	2720	639	23	-
North Sumatra	3	100	74	59 (1.4–116.7)	99.09
West Sumatra	3	138	90	51.6 (22.9–80.2)	88.39
South Sumatra	1	221	173	78	-
Riau	3	427	322	77.6 (52.1–103.2)	98.57
Bangka Belitung	2	42	14	28.6 (0.6–55.6)	78.57
Lampung	2	101	58	51.2 (25–77.5)	81.44
Banten	5	538	290	45.6 (24.2–67.1)	96.69
West Java	4	630	242	28 (8.2–47.8)	96.64
Central Java	9	2753	1024	44.5 (31.5–57.4)	98.2
East Java	25	3693	1597	44.1 (27.8–60.4)	99.52
Yogyakarta	6	621	364	55.9 (38.8–72.9)	94.31
Central Kalimantan	3	224	163	73 (67.2–78.8)	NS
Central Indonesia					
East Kalimantan	2	73	62	85.4 (77.4–93.5)	NS
North Kalimantan	1	19	7	37	-
Bali	11	1794	635	37.8 (19.6–55.9)	99.13
West Nusa Tenggara	6	500	340	66.6 (55–78.2)	86.87
East Nusa Tenggara	2	118	51	37.6 (−20–95.2)	98.62
Southeast Sulawesi	3	165	65	48.3 (17–79.5)	92.41
West Sulawesi	3	75	34	43.7 (14.7–72.7)	86.67
South Sulawesi	4	335	242	49.5 (12.4–86.7)	97.79
Central Sulawesi	4	145	91	48.8 (10–87.5)	97.12
Gorontalo	1	30	10	33	-
Eastern Indonesia					
North Maluku	2	51	34	53.2 (−27.5–133.9)	98.86
Papua	3	175	83	46.6 (5–88.3)	97.92
West Papua	4	990	522	51 (43–58.9)	78.67

NS=Not significant

## Discussion

GIPs cause diseases that affect livestock worldwide. Although infected livestock often do not manifest noticeable clinical symptoms, the economic impact of such infections is substantial. These parasites can cause blood loss, tissue damage, spontaneous abortion, and reduced host immunity, ultimately affecting the body weight of livestock [19–21,74].

The pooled estimates demonstrated a 46% prevalence of bovine GIPs in Indonesia, similar to the prevalence reported in India (47%). Nematodes constitute India’s largest proportion of parasites, accounting for 24% of all parasites [75]. Protozoa play a significant role in gastrointestinal infections in cattle in Indonesia due to their direct life cycle. In contrast, trematodes and cestodes exhibit lower prevalence rates due to their more complex life cycles that require intermediate hosts [10, 22]. The cattle-infecting protozoa included *Eimeria, Blastocystis, Entamoeba*, and *Balantidium* species, highlighting the diversity of protozoan infections in cattle populations [1, 19]. The prevalence of these protozoan infections, particularly *Eimeria* and *Blastocystis*, has been documented in different regions of Indonesia, emphasizing the need for effective control measures to manage these infections [1, 15, 23–26].

The prevalence of strongyle-type nematodes was higher in Indonesia than Egypt and India, with a pooled estimate of 30.8%. The prevalence of *Trichostrongyle* in cattle was reported as 27.4% in Egypt [76], whereas Krishnamoorthy *et al*. [75] reported a 24% prevalence of nematode infection in India. The high prevalence of strongyle-type nematodes in Indonesia could be attributed to climate, management practices, and host susceptibility. Nematodes pose a significant threat to livestock health and productivity in regions where they are widespread.

The harmful effects of liver fluke infestation on cattle health and productivity in Indonesia have been well documented in various studies on *F. gigantica* infestations [3, 22, 27]. Fluke infection significantly and negatively affects daily, live, and carcass weights [77]. Prevalence of *Fasciola* in Indonesia was 21.6%, compared with 13.84% in Nigeria [78].

The use of animal manure as fertilizer in agricultural practices can contaminate land with infectious larvae. Moreover, the climatic conditions in Indonesia, which are characterized by high temperatures and humidity, provide an ideal environment for larval development and survival. Furthermore, many owners lack awareness of the economic implications of these parasites and their transmission routes, which contribute to their spread. In Indonesia, many farmers clean cattle pens but often pile feces around the pens and remove it once a large amount of feces has accumulated, facilitating disease transmission to nearby cows.

The meta-analysis included two 5-point publications and four 4-point articles, in which most studies scored one. Most of the missing articles were due to missing detailed geographical coordinates of the sampling location, <200 tests, and no analytical risk factors. Thus, future studies with larger sample sizes are needed to analyze other risk factors. Moreover, researchers should investigate additional factors associated with GIPs. Comprehensive research on the prevalence and risk factors of GIP provides epidemiological data and a theoretical foundation for parasite control programs.

We observed substantial heterogeneity among studies reporting bovine GIP infections in cattle, which persisted even after species-specific and geographical analyses. The absence of publication bias, as demonstrated by the funnel plots and Egger’s regression, suggests that the results of the present study can be used to make decisions regarding parasite infection management in Indonesia. Sensitivity analysis revealed that no single study changed the pooled prevalence.

Subgroup analysis by region revealed that studies on GIPs in Indonesia have been predominantly conducted in the Western region, primarily due to the concentrated cattle populations in areas such as Java. The pooled prevalence indicated that East Java had the highest prevalence of parasites. Considering the significant number of studies conducted in East Java and its status as the province with the largest cattle population, special efforts are required to control parasitic infections [4]. Further studies are needed nationwide, particularly in regions with significant cattle populations and areas where no prior epidemiological studies have investigated bovine GIPs using conventional and molecular approaches.

The inconsistent methodologies employed across Indonesia may hinder the comparison of prevalence data between regions. Traditional methods are the most widely used in Indonesia and include the flotation of nematode eggs, cestode eggs, and protozoan cysts, as well as the sedimentation of trematode eggs. However, variations exist in flotation techniques such as Whitlock, McMaster, Sugar Flotation, and FLOTAC. Microscopy remains essential for parasitological diagnostics, especially in field and low-resource settings, and provides crucial epidemiological assessments of parasite burdens [79]. However, these approaches are time-consuming, labor-intensive, and highly dependent on the technical expertise of laboratory personnel. Coprological techniques also have limitations, such as poor sensitivity and the inability to differentiate closely related species [80]. Molecular identification based on ribosomal internal transcribed spacer regions is crucial for identifying specific parasites, such as *Fasciola hepatica* and *Eimeria* spp. [21, 81]. Advances in multiplex PCR techniques have facilitated the detection and differentiation of various *Eimeria* spp. in cattle, highlighting the need for DNA-based technologies in diagnostic laboratories [25].

The use of anthelmintics for treating parasitic diseases in cattle varies depending on the specific parasite. Commonly used anthelmintic drugs targeting GIPs in cattle include albendazole, ivermectin, levamisole, and fenbendazole [28]. These drugs are effective against various parasites, including helminths and protozoans. Strategic deworming plays an essential role in controlling parasites in cattle. This involves creating a deworming schedule tailored to specific factors, such as cattle age, parasite prevalence in the area, and seasonal variations in parasite burden [29]. Strategic deworming, when performed at optimal times with appropriate anthelmintics, can maximize the efficacy of parasite control and reduce the risk of developing drug resistance.

## Conclusion

The prevalence of bovine GIPs in Indonesia varies across regions and remains poorly documented. The complexity of these infections, which are influenced by species diversity, genetic variability, and environmental conditions, underscores the importance of tailored control strategies and ongoing research to mitigate the impact of these parasites on livestock health. However, due to the limited availability of relevant data, obtaining a comprehensive understanding of the factors influencing the prevalence of parasitic infections on a national scale is challenging. Appropriate preventive measures require knowledge of the distribution and prevalence of GIPs in cattle in Indonesia, as well as standardization of detection methods.

## Authors’ Contributions

VIN and FE: Conducted the literature search, identified and screened articles, extracted data, and discussed about the selection of articles with JP and WN. VIN and FE: Conducted statistical and meta-analyses and drafted the manuscript. JP, IW, and WN: Supervised and verified the screening and selection of articles and revised the manuscript. All authors have read and approved the final version of the manuscript.
